# Evaluation of the Efflux Pump Inhibition Activity of Thiadiazine-Derived Compounds Against the *Staphylococcus aureus* 1199B Strain

**DOI:** 10.3390/ph18030323

**Published:** 2025-02-25

**Authors:** Cicera Laura Roque Paulo, Priscilla Ramos Freitas Alexandre, Ana Carolina Ferreira Araujo, Ray Silva Almeida, Emílio Sousa Albuquerque, Cícera Datiane de Morais Oliveira-Tintino, Igor J. S. Nascimento, João Xavier Araújo-Júnior, Edeildo Ferreira da Silva-Junior, Thiago Mendonça de Aquino, Francisco Jaime Bezerra Mendonça-Junior, José Bezerra de Araújo-Neto, Maria Karollyna do Nascimento Silva Leandro, Irwin Rose Alencar de Menezes, Henrique Douglas Melo Coutinho, Janaina Esmeraldo Rocha

**Affiliations:** 1 Laboratory of Microbiology and Molecular Biology—LMBM, Regional University of Cariri—URCA, Crato 63105-000, CE, Brazil; laura.roque@urca.br (C.L.R.P.); priscilla.freitas@urca.br (P.R.F.A.); caroljustino@outlook.com (A.C.F.A.); rayalmeidasilva2306@gmail.com (R.S.A.); emilioalbuq@gmail.com (E.S.A.); datianemorais@gmail.com (C.D.d.M.O.-T.); karollyna.silva@urca.br (M.K.d.N.S.L.); janainaesmeraldo@gmail.com (J.E.R.); 2 Laboratory of Medicinal Chemistry, Institute of Pharmaceutical Sciences, Federal University of Alagoas, Maceió 57072-900, AL, Brazil; igorjsn@hotmail.com (I.J.S.N.); jotaaraujo2004@gmail.com (J.X.A.-J.); 3 Biological and Molecular Chemistry Research Group, Institute of Chemistry and Biotechnology, Federal University of Alagoas, Maceió 57072-900, AL, Brazil; edeildo.junior@iqb.ufal.br; 4 Laboratory of Synthesis and Research in Medicinal Chemistry, Research Group on Therapeutic Strategies—GPET, Institute of Chemistry and Biotechnology, Federal University of Alagoas, Maceió 57072-900, AL, Brazil; thiago.aquino@iqb.ufal.br; 5 Laboratory of Synthesis and Drug Delivery—LSVM, Paraiba State University, João Pessoa 58071-160, PB, Brazil; franciscojbmendonca@yahoo.com.br; 6 Postgraduate Program in Biological Sciences, Biosciences Center, Federal University of Pernambuco, Recife 50740-570, PE, Brazil; jose.bezerra456@gmail.com; 7 Laboratory of Pharmacology and Molecular Chemistry—LFQM, Regional University of Cariri—URCA, Crato 63105-000, CE, Brazil

**Keywords:** antibiotic resistance, thiadiazines, *Staphylococcus aureus*

## Abstract

**Background:** Substances with antibacterial properties have become crucial in light of the continuous increase in infections caused by multidrug-resistant bacteria. In this context, thiadiazines have emerged as heterocyclic compounds already known for their pharmacological activities. However, their potential as antibacterial agents and inhibitors of the efflux system found in resistant bacteria remains poorly understood. From this perspective, the present study highlights the synthesis of thiadiazine-derived compounds and evaluates their antibacterial activity and efflux pump inhibition against the *Staphylococcus aureus* 1199B strain. **Methods:** To this end, Minimum Inhibitory Concentration (MIC) tests were conducted, along with the analysis of antibacterial activity through the inhibition of the NorA efflux system using 96-well microdilution assays. Additionally, to assess efflux system inhibition, ethidium bromide (EtBr) fluorescence emission tests were performed, alongside in silico molecular docking studies. **Results:** Based on the results obtained, it was observed that compound IJ28 exhibited direct activity against the tested SA 1199B strains, with an MIC of 512 µg/mL. It also demonstrated antibacterial activity through efflux pump inhibition, resulting in increased fluorescence rates emitted by EtBr. Compound IJ28 showed a more significant reduction in the Minimum Inhibitory Concentration (MIC) of ethidium bromide, decreasing from 26.6 µg/mL to 0.5 µg/mL, compared to the other compounds. **Conclusions:** Therefore, it is essential to conduct further studies to investigate the mechanism of action and clarify the feasibility and effects of compound IJ28 as a potential antibacterial agent.

## 1. Introduction

Described as a member of the normal human microbiota, the bacterium *Staphylococcus aureus* is a Gram-positive species with a cocci-shaped morphology. It is considered one of the most important pathogenic bacteria, as it acts as an agent of a wide range of infections, varying from localized to systemic infections, the latter being of high severity. Its clinical importance has significantly increased, mainly due to the rising incidence of severe hospital-acquired infections caused by multidrug-resistant strains, such as MRSA (Multi-Resistant *S. aureus*) [1,2].

The adaptive capacity of *S. aureus* to antibiotics is a significant public health concern.* This adaptability can occur in various ways, as it is known to develop tolerance through both intrinsic genetic mechanisms and the acquisition of defense genes. The main resistance mechanisms of *S. aureus* include the following: 1. Production of inactivating enzymes; 2. Target alterations; 3. Efflux mechanisms [3,4,5].

Efflux mechanisms play essential physiological roles in bacterial cells, encompassing both the influx and efflux of various components. However, these microorganisms have exploited this system as a resistance strategy, actively promoting the efflux of antibiotics and reducing their intracellular concentrations [6].

A notable example is the NorA efflux pump, encoded by the norA gene, which is part of the major facilitator superfamily (MFS) commonly found in bacteria such as *S. aureus*. This transporter protein plays a vital role in expelling antimicrobial agents, including hydrophobic fluoroquinolones and various biocides [7,8,9,10,11].

The synthesis of compounds represents a significant approach as an alternative source for microbial control across various fields, ranging from medicine to industry. In the pharmaceutical sector, the synthesis of new antimicrobial compounds is vital due to the growing challenge of antibiotic resistance. Researchers aim to develop compounds that are effective against pathogenic microorganisms while also minimizing environmental impact, given the global concern about antimicrobial resistance and the preservation of ecosystems [12,13,14].

In the chemical synthesis of thiadiazine derivatives as an alternative approach, the process involves several steps, including the selection of suitable precursors, specific chemical reactions, and purification of intermediate products. The chemical structure must be carefully designed to ensure the compound’s effectiveness in efflux system inhibition while minimizing potential adverse effects [12].

Thiadiazines constitute a class of six-membered heterocyclic compounds characterized by the presence of two nitrogen atoms and one sulfur atom in their molecular structure. These compounds play a significant role in medicinal chemistry, exhibiting various properties such as antifungal, antimicrobial, antiviral, antioxidant, and anticancer activity.

The identification of compounds that act synergistically with antibiotics, such as norfloxacin, may enable new strategies for the treatment of resistant infections. The ability of these compounds to modulate the bacterial efflux system is of particular interest, as the efflux system is a crucial resistance mechanism in many bacterial strains. This study aims to evaluate, through in vitro and in silico methods, the ability of these compounds to inhibit the NorA efflux pump in *Staphylococcus aureus*, a key resistance mechanism in various strains.

## 2. Results

Following the synthesis procedure, four solid compounds with a yellow coloration were obtained, and their characteristics are described below: The compound 5-(4-methoxyphenyl)-N-phenyl-6H-1,3,4-thiadiazin-2-amine, identified as **IJ26**, was obtained with a yield of 56% and exhibited a melting point in the range of 187–188 °C.

The compound 5-(4-chlorophenyl)-N-phenyl-6H-1,3,4-thiadiazin-2-amine, identified as **IJ27**, was isolated with a yield of 60% and showed a melting point between 179 and 180 °C.

The compound 4-(2-(phenylamino)-6H-1,3,4-thiadiazin-5-yl)phenol, identified as **IJ28**, was obtained with a yield of 60% and a melting point ranging from 111 to 112 °C.

Finally, the compound N-phenyl-5-(p-tolyl)-6H-1,3,4-thiadiazin-2-amine, identified as **IJ29**, presented a yield of 50% and a melting point in the range of 180–181 °C.

These results highlight the efficiency of the syntheses and the consistency in melting points, indicative of well-defined and purified compositions.

### 2.1. Antibacterial Activity

**Figure 1:**
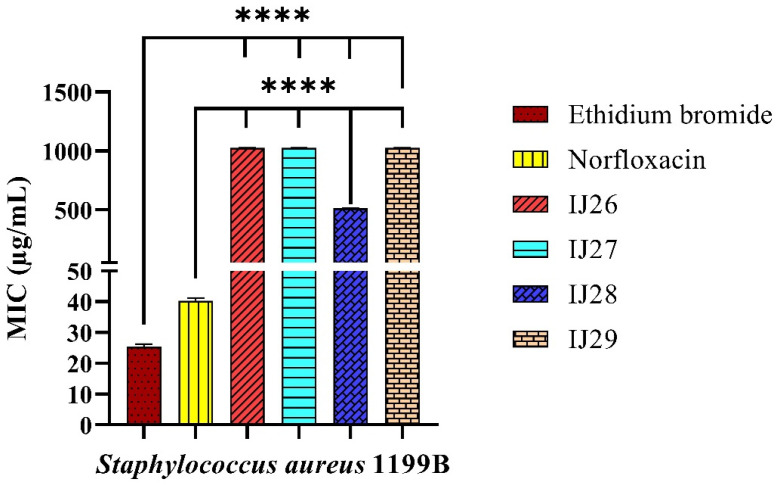
Activity of thiadiazine-derived compounds tested in combination with ethidium bromide and norfloxacin against *Staphylococcus aureus* 1199B strains. **** *p* < 0.0001 indicates significant differences between the groups. Statistical significance was determined by one-way ANOVA and Bonferroni post hoc test.

By implementing the described methodology, it was observed that, among the tested derivative compounds, only the product IJ28 exhibited direct activity against the tested SA 1199B strain, with a MIC of 512 µg/mL. The other analyzed compounds, IJ26, IJ27, and IJ29, showed no antibacterial activity, with MIC values ≥ 1024 µg/mL. These results are presented in Figure 1, which illustrates the distribution of MIC values for the tested compounds.

### 2.2. Evaluation of Antibacterial Activity Through Efflux Pump Inhibition

The activity of the efflux pump in the bacterial strain under analysis can be examined using ethidium bromide and Carbonyl Cyanide m-Chlorophenylhydrazone (CCCP). CCCP is a cellular membrane uncoupler that dissipates the proton gradient, essential for the functioning of bacterial efflux pumps. This prevents the expulsion of substances such as ethidium bromide and norfloxacin, allowing their intracellular accumulation, which facilitates the analysis of the activity of these pumps through the fluorescence of ethidium bromide.

Chlorpromazine (CPZ) directly inhibits efflux pumps, such as the NorA protein, blocking the expulsion of antibiotics and leading to the retention of ethidium bromide and norfloxacin within the cells. This effect increases the antibiotic’s efficacy and allows the study of bacterial resistance modulation. Both compounds, CCCP and CPZ, are essential for understanding efflux inhibition and the intracellular accumulation of substances. In this context, the sub-inhibitory concentration of the compound was combined with ethidium bromide and the antibiotic, resulting in a reduction in the Minimum Inhibitory Concentration (MIC).

**Figure 2:**
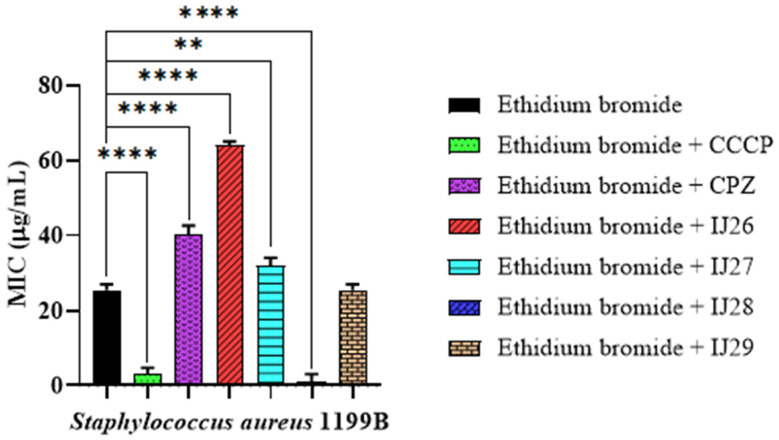
Activity of thiadiazine-derived compounds tested in combination with ethidium bromide and CCCP against *Staphylococcus aureus* 1199B strains. **** *p* < 0.0001 and ** *p* < 0.01 indicates significant differences between the groups. Statistical significance was determined by one-way ANOVA and Bonferroni post hoc test.

The results of the combination of compounds with ethidium bromide indicate that there was no efflux pump expression, as observed in Figure 2. The compound IJ28 demonstrated a more significant reduction in the Minimum Inhibitory Concentration (MIC) of ethidium bromide, decreasing from 26.6 µg/mL to 0.5 µg/mL, compared to the other compounds, resulting in the inhibition of the norA efflux pump.

Comparing the corresponding thiadiazine compounds evaluated in combination with the antibiotic norfloxacin, the products IJ26 and IJ27 did not show relevant activity that could indicate potential antagonism. A possible explanation for this observation is that these compounds may be inducing or increasing the expression of bacterial efflux pumps, such as the NorA protein in *Staphylococcus aureus*. Efflux pumps are systems that expel toxic or antimicrobial substances from the cell, reducing the intracellular concentration of antibiotics and other compounds, which may lead to an increase in MICs.

To test this hypothesis, experiments could be conducted to assess the gene expressi-on of efflux pumps in the presence of compounds IJ26 and IJ27, using techniques such as real-time PCR to quantify the gene NorA levels of efflux proteins.

**Figure 3:**
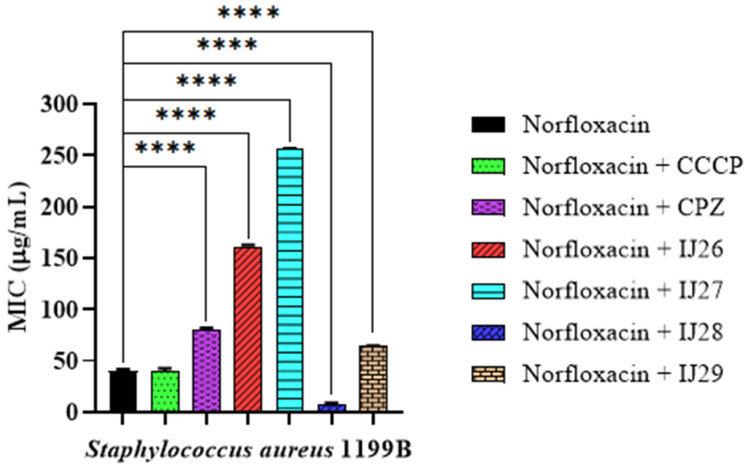
Activity of thiadiazine-derived compounds tested in combination with the antibiotic norfloxacin against *Staphylococcus aureus* 1199B strains. **** *p* < 0.0001 indicates significant differences between the groups. Statistical significance was determined by one-way ANOVA and Bonferroni post hoc test.

On the other hand, the compound IJ28, when combined with the antibiotic, showed a significant reduction in the Minimum Inhibitory Concentration (MIC) from 42.6 µg/mL to 8 µg/mL, as illustrated in Figure 3.

### 2.3. Evaluation of NorA Efflux Pump Inhibition by Fluorescence Emission

**Figure 4:**
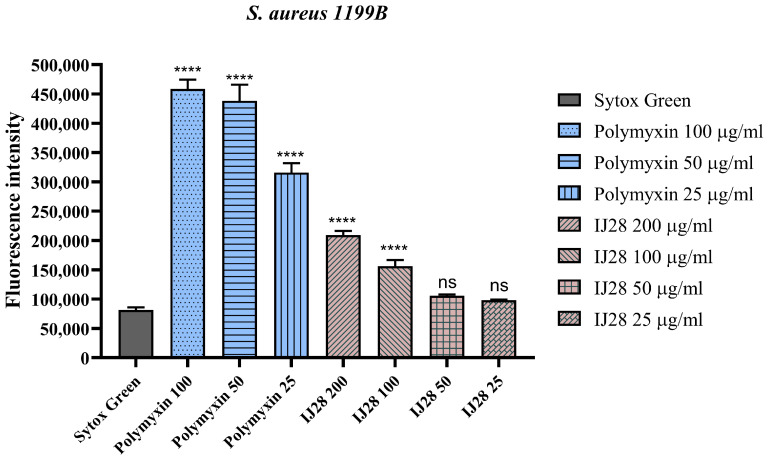
Evaluation of NorA efflux pump inhibition by measuring fluorescence emission in *S. aureus* 1199B strains treated with IJ28 at concentrations of 20 and 50 µg/mL. EtBr = ethidium bromide; ns = not significant; **** = *p* < 0.0001 vs. EtBr.

When evaluating the fluorescence emission in the *S. aureus* 1199B strain, it was observed that the sample containing IJ28 at concentrations of 20 and 50 µg/mL caused a significant increase in the fluorescence emission of EtBr by 30% and 21%, respectively, compared to the control with EtBr alone (Figure 4). Ethidium bromide is used to demonstrate the activity of the efflux pump since, unlike antibiotics, its elimination from the cell occurs exclusively through this mechanism. Thus, its intracellular detection confirms the presence and functionality of the pump. The *Staphylococcus aureus* 1199B strain was chosen because it is a well-known mutant variant that overexpresses the *norA* gene, which is responsible for the efflux of fluoroquinolones. The IJ28 compound was tested individually and did not exhibit fluorescence detectable by the equipment.

### 2.4. Molecular Docking Evaluation of Compound IJ28

Among the evaluated derivatives, compound IJ28 exhibited the lowest values in the three parameters used: binding energy (−5.55 Kcal/mol), inhibition constant (84.96 µM), and ligand efficiency (−0.28). These results indicate IJ28 as the most effective in silico, supporting the in vitro assay results. Furthermore, this derivative proved to be more efficient compared to CCCP, as described in the previous sections, except in ligand efficiency due to the lower number of heavy atoms (non-hydrogen atoms) in the efflux inhibitor molecule (Table 1).

**Figure 5:**
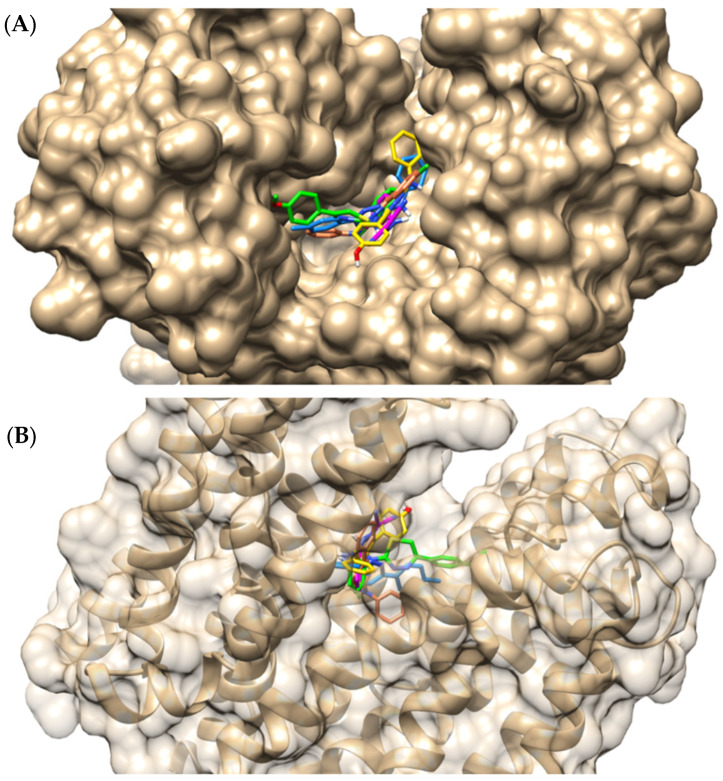
Top (**A**) and side (**B**) view of the location of the compounds in the NorA binding site: IJ26—green, IJ27—orange, IJ28—yellow, IJ29—blue, CCCP—magenta.

**Figure 6:**
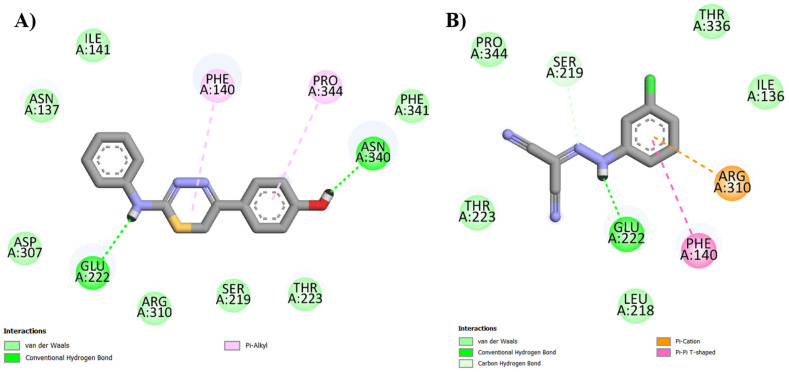
Interactions between IJ28 (**A**) and CCCP (**B**) and the NorA efflux pump.

As demonstrated in Figure 5, portions of the thiadiazine derivatives and CCCP molecules occupy the same region in the protein cavity. However, of the four derivatives, IJ28 is the compound with the position most similar to that of the reference inhibitor. This information is corroborated by the interactions that IJ28 and CCCP performed with the efflux pump, such as hydrogen bonding with Glu222 and hydrophobic interactions with Phe140 (π-alkyl and π-π T-shaped, respectively), in addition to interacting in different ways with identical residues, e.g., Pro344 and Ser219 (Figure 6, Table 2).

## 3. Discussion

According to reports in the literature, studies involving triazoles have shown activity against the SA 1199B strain, leading to the inhibition of bacterial growth. The reduction in the Minimum Inhibitory Concentration of the compounds was demonstrated by the products IJ26, IJ27, IJ28, and IJ29, a mechanism that can be explained by the blocking of the efflux system. The lack of direct activity of compounds IJ26, IJ27, and IJ29 may be attributed to a lower affinity for essential bacterial targets or challenges in membrane permeability, a factor previously reported for other classes of heterocyclic compounds [15,16].

Furthermore, the literature indicates that the overexpression of the NorA efflux pump in *S. aureus* 1199B is a key factor in reducing the efficacy of various antibiotics, including norfloxacin [9].

Studies by Pereira (2020) [16,17] suggest that thiadiazine-derived compounds appear to exhibit synergistic activity when combined with the antibiotic norfloxacin, resulting in a significant decrease in the minimum inhibitory concentration against the 1199B bacterial strain.

These findings suggest that the modulation of the antibacterial activity of thiadiazine derivatives may be related to specific structural factors, as previously described in the literature [15,17].

In recent years, studies on thiazole-derived compounds have garnered significant interest among researchers due to their potential biological activities. As reported, some of these compounds have proven effective in the treatment of various conditions infections, further encouraging research and the development of new drugs based on these compounds [15,16,18,19].

In the evaluation of efflux system inhibition in bacteria carrying the norA gene, microdilution tests were performed using fluorimetric analysis, where ethidium bromide (EtBr) acted as a DNA intercalator, emitting fluorescent light upon binding to genetic material. Consequently, the reduced functionality of the efflux protein leads to an increased concentration of EtBr within the cells [20]. A notable increase in fluorescence was observed when using thiadiazine-derived compounds, indicating potential inhibition of the proper function of the NorA efflux pump.

According to research conducted by De Araújo and collaborators (2023) [16], the efficacy of limonene as an efflux pump inhibitor in *Staphylococcus aureus* RN4220 strains was demonstrated. Molecular docking showed that, in addition to the affinity between thiadiazine derivatives and the efflux pump, IJ28 and CCCP interact with common residues, reinforcing that the evaluated compounds modulate resistance to norfloxacin through NorA inhibition. Residues identified in our study, such as Phe140 and Asn340, were also targeted by reserpine, known for its inhibitory effect on efflux pumps [21]. Brawley et al. (2022) [22] support our findings by describing key residues for NorA activity and, therefore, crucial for its inhibition, including Asn137, Phe140, Glu222, and Asp307.

A study demonstrated that phenothiazine dyes acted as substrates for multidrug-resistant efflux pumps in bacteria. This finding was obtained by employing the compound NorA (INF271 diphenylurea, reserpine, 5′-methoxy-hydrocarnoquinone, and polyalkylated neohesperidose, ADH7) in combination with red light in *S. aureus* [23,24,25].

## 4. Materials and Methods

### 4.1. Material Acquisition

Chemicals and solvents were obtained from Sigma-Aldrich^®^ (Saint Louis, MO, USA) and used without further purification. All reactions were monitored using thin-layer chromatography (TLC) on aluminum plates coated with Merck Kieselgel 60 F254 silica gel and visualized under ultraviolet light (257 nm). The compounds were purified on Merck^®^ 60 silica gel (particle size 0.040–0.063 nm) or by using flash chromatography with Armen^®^ spot chromatography (normal-phase column: Interchim 30 SHIP 25 g; reverse-phase column: AIT 50 g C18).

^1^H and ^13^C NMR spectra were recorded on a Bruker^®^ Avance III Spectrometer (Karlsruhe, Germany)operating at 600 MHz and 150 MHz, respectively. Chemical shifts were reported in δ units, and coupling constants (J) were measured in hertz. The peaks are presented as s (singlet), d (doublet), t (triplet), q (quartet), quint (quintet), br s (broad singlet), dd (double doublet), td (triplet of doublets), and m (multiplet). Melting points were determined using the MSTecnopon^®^ PFMII melting point apparatus and were not corrected. The purity of all compounds was determined by HPLC (Shimadzu SIL-20AHT, Kyoto, Japan) with a Supelco Discovery C-18 column and methanol, methanol/formic acid, methanol/water, or water as the mobile phase.

### 4.2. General Procedure

A mixture of the required thiosemicarbazide (1 mmol) and 2-bromoacetophenone (1 equiv) in 15–20 mL of an appropriate solvent (MeOH) was stirred at room temperature and refluxed until complete conversion of the starting material, for 1 h. The reaction mixture was then allowed to stand at room temperature, and the separated solid product was filtered (Box 1, Box 2, Box 3 and Box 4). The compounds were purified by recrystallization from methanol/water.

Box 1 **Synthesis characterization, yield and spectroscopic data of IJ26.**

**5-(4-Methoxyphenyl)-N-phenyl-6H-1,3,4-thiadiazin-2-amine IJ26** Yellow solid; yield 56%; mp 187–188 °C; HPLC-UV: 3.07 min.; purity of 99%; ^1^H NMR (600 MHz, DMSO-d6) δ 3.85 (s, 3H, OCH_3_); 4.30 (s, 2H, CH_2_); 7.12 (dt, 2H, J = 3.10 and 8.95 Hz, H-Ar); 7.34 (t, 1H, J = 7.38 Hz, H-Ar); 7.45–7.50 (m, 4H, H-Ar); 7.92 (dt, 2H, J = 3.10 and 8.95 Hz, H-Ar); ^13^C NMR (150 MHz, DMSO-d6) δ 162.2; 154.4; 151.5; 137.7; 129.4; 129.2; 126.7; 123.9; 123.3; 114.5; 114.1; 55.5; 22.7.

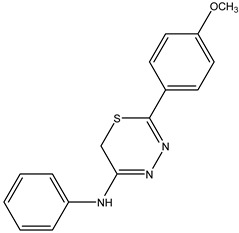


**IJ26**

Box 2 **Synthesis characterization, yield and spectroscopic data of IJ27.**

***5-(4-Chlorophenyl)-N-phenyl-6H-1,3,4-thiadiazin-2-amine*—IJ 27** Yellow solid; yield 60%; mp 179–180 °C; HPLC-UV: 3.27 min.; purity of 99%; ^1^H NMR (600 MHz, DMSO-d6) δ 3.74 (s, 2H, CH_2_); 7.06 (dd, 2H, J = 1.18 and 8.62 Hz, H-Ar); 7.18 (tt, 1H, J = 1.18 and 7.46 Hz, H-Ar); 7.37 (dtq, 2H, J = 1.95, 8.62 and 0.76 Hz, H-Ar); 7.43 (dt, 2H, J = 2.58 and 8.70 Hz, H-Ar); 7.71 (dt, 2H, J = 2.58 and 8.70 Hz, H-Ar); ^13^C NMR (150 MHz, DMSO-d6) δ 153.4; 145.9; 145.5; 136.1; 133.0; 129.1; 129.0; 127.4; 124.7; 122.4; 23.2.

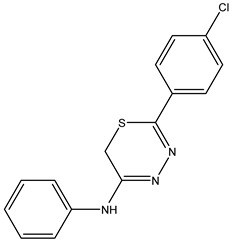


**IJ 27**

Box 3 **Synthesis characterization, yield and spectroscopic data of IJ28.**

***4-(2-(Phenylamino)-6H-1,3,4-thiadiazin-5-yl)phenol*—IJ28** Yellow solid; yield 60%; mp 111–112 °C; HPLC-UV: 2.85 min.; purity of 99%; ^1^H NMR (600 MHz, DMSO-d6) δ 3.82 (s, 2H, CH_2_); 6.82 (dt, 3H, J = 2.86 and 8.86 Hz, H-Ar); 7.01 (t, 1H, J = 7.46 Hz, H-Ar); 7.28 (dt, 3H, J = 1.84 and 8.01 Hz, H-Ar); 7.70 (s, 2H, H-Ar); 9.87 (s, 1H, NH); 10.12 (s, 1H, OH); ^13^C NMR (150 MHz, DMSO-d6) δ 159.3; 147.2; 129.1; 128.2; 126.4; 123.1; 122.0; 116.5; 115.9; 115.5; 22.6.

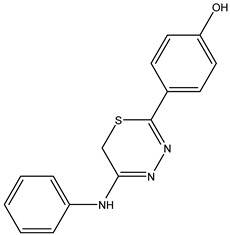


**IJ 28**

Box 4 **Synthesis characterization, yield and spectroscopic data of IJ29.**

***N-phenyl-5-(p-tolyl)-6H-1,3,4-thiadiazin-2-amine*—IJ29** Yellow solid; yield 50%; mp 180–181 °C; HPLC-UV: 3.18 min.; purity of 99%; ^1^H NMR (600 MHz, DMSO-d6) δ 2.39 (s, 3H, CH_3_); 4.29 (s, 2H, CH_2_); 7.35–7.38 (m, 3H, H-Ar); 7.42–7.43 (m, 2H, H-Ar); 7.50 (t, 2H, J = 8.09 Hz, H-Ar); 7.84 (d, 2H, J = 8.3 Hz, H-Ar); ^13^C NMR (150 MHz, DMSO-d6) δ 142.3; 130.6; 130.1; 130.0; 129.2; 127.7; 127.6; 124.8; 123.9; 23.1; 21.5. ^13^C NMR (150 MHz, DMSO-d6) δ 159.3; 147.2; 129.1; 128.2; 126.4; 123.1; 122.0; 116.5; 115.9; 115.5; 22.6.

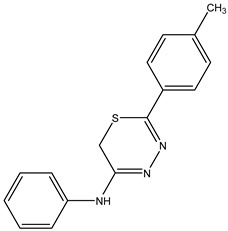


**IJ 29**

### 4.3. Preparation of Microorganisms and Inoculum for Microdilution

The *Staphylococcus aureus* 1199B strain used was provided by the University of London. For the experiment, the strains were inoculated into Brain Heart Infusion (BHI) culture medium on agar plates and incubated for 24 h at 37 °C. After this period, the bacterial inoculum was prepared in test tubes containing sterile saline solution, and an aliquot of bacteria was added to reach a McFarland scale value of 0.5, corresponding to 10^8^ CFU/mL.

### 4.4. Preparation of Substances for Microdilution Test

The components used were chlorpromazine (CPZ), ethidium bromide, m-chlorophenylhydrazone cyanide (CCCP), and the antibiotic norfloxacin; all of these products were purchased from Sigma-Aldrich, St. Louis, MO, USA. For the dilution of ethidium bromide, 10 mg was weighed, followed by the addition of 9.765 µL of sterile distilled water. For the CCCP dilution, the material was weighed and diluted with 9.765 µL of a previously prepared solution containing 5 mL of methanol and 5 mL of sterile distilled water. For the preparation of the antibiotic and the thiazine-derived compounds, 10 mg of each substance was weighed, and subsequently, 500 µL of dimethyl sulfoxide (DMSO) was added, followed by 9.265 µL of sterile distilled water. All products were prepared with an initial concentration of 1024 µg/mL.

### 4.5. Determination of Minimum Inhibitory Concentration (MIC)

For the determination of the Minimum Inhibitory Concentration (MIC), a solution was initially prepared in microtubes with a total volume of 1000 µL. This solution contained 100 µL of bacterial inoculum, corresponding to 10% of the total solution volume, and the remaining 900 µL consisted of 10% BHI (Brain Heart Infusion) medium. From the prepared solution, 100 µL was transferred to each well of the 96-well microdilution plates and then microdiluted with 100 µL of the products up to the second-to-last well. The dilutions ranged from a concentration of 512 to 8 µg/mL. The last well was used as a positive control. Subsequently, the plates were incubated in an oven at 37 °C for 24 h. A negative control was also performed, involving the transport of 10% BHI medium to the wells, followed by incubation. The entire test was performed in triplicate. For the interpretation of the results, 20 µL of sodium resazurin was added and maintained at room temperature for 1 h. During this time, a color change from blue to pink was observed, indicating bacterial growth [26].

### 4.6. Evaluation of Antibacterial Activity Through Efflux Pump Inhibition

The evaluation of efflux pump inhibition was carried out by performing an experiment using the sub-inhibitory concentration of the compounds (MIC/8). In a microtube, 150 µL of bacterial inoculum, representing 10% of its total capacity, was added, and then a volume corresponding to the sub-inhibitory concentration of the product was added to the tubes, which were completed to a total volume of 1500 µL using 10% BHI medium. After filling the tubes, the solution was transferred to sterile 96-well microplates, and a serial dilution was performed with 100 µL of the products, ranging from an initial concentration of 512 µg/mL to 0.5 µg/mL. Subsequently, the microplates were incubated at approximately 37 °C for 24 h. The results were interpreted by adding 20 µL of sodium resazurin. Controls containing the minimum inhibitory concentrations (MIC) of antibiotics and ethidium bromide were used. All tests were performed in triplicate [27].

### 4.7. Inhibitory Action of Efflux Pumps NorA Assessed by Increased Fluorescence Emission of Ethidium Bromide

The *Staphylococcus aureus* 1199B strains were cultured in Mueller Hinton agar 24 h prior to the test and then incubated in a 37 °C incubator. The bacterial inoculum was prepared in phosphate-buffered saline (PBS) to achieve a colony-forming unit count of 1.5 × 10^8^, according to the McFarland scale 0.5. Sample solutions containing bacterial inoculum and IJ28 at concentrations of 20 µg/mL or 50 µg/mL were prepared. CCCP at 50 µg/mL was used as a positive control. The solutions were incubated for 1 h and 30 min. Ethidium Bromide (EtBr) at 100 µg/mL was then added to all solutions except the growth control group (only inoculum). The samples remained in the incubator for 1 h. Subsequently, the solutions were centrifuged at 10,000 rpm for 2 min and washed with PBS, discarding the supernatant, until EtBr and residual substances were removed. The pellet was dissolved in PBS and distributed into microplates. The readings were performed using a BioTek^®^ Cytation 1 fluorescence microplate reader (Agilent Technologies, Santa Clara, CA, USA) and the Gen5™ 3.11 software with excitation at 530 nm and emission wavelength at 590 nm. Readings were conducted for the following groups: inoculum only (growth control); inoculum + EtBr (negative control); inoculum + EtBr + CCCP at 50 µg/mL (positive control); inoculum + EtBr + IJ28 at concentrations of 20 µg/mL or 50 µg/mL. The assay was performed in triplicate [22].

### 4.8. Molecular Docking Evaluation of Compound IJ28

The model of the NorA efflux pump was constructed using SWISS-MODEL [28] with the available sequence from UniProt (entry Q03325) and the corresponding model from PDB identifier 7lo8, following the study by Araújo-Neto et al. (2023) [29]. We also used the coordinates of the binding site center (x, y, z) = (140.37, 137.26, 150.16). The structures of the thiazine derivatives and CCCP were built following the method described by Dos Santos Barbosa et al. (2023) [25]. AutoDockTools v. 1.5.7 [30] was used to prepare ligands and receptors, where hydrogens were added (and non-polar hydrogens merged), Gasteiger charges were applied, and flexibility was incorporated. In receptor flexibility, the relevant residues described by Brawley et al. (2022) [22] were considered.

The flexible docking software used was AutoDock v. 4.2.6 (https://autodock.scripps.edu/; accessed on 4 May 2024). The grid box (30 Å × 30 Å × 30 Å) was positioned at the center of the selected binding site. For the search parameters, the Lamarckian Genetic Algorithm was applied with a total of 100 runs, a population size of 150, a maximum of 2,500,000 evaluations, a maximum of 27,000 generations, and a root mean square deviation (RMSD) tolerance of 2.0 Å. The conformations with the lowest binding energies were analyzed using ChimeraX v 1.9 [31] and BIOVIA Discovery Studio 2021 v. (https://www.3ds.com/products/biovia/discovery-studio/, accessed on 4 May 2024).

### 4.9. Statistical Analysis

For statistical analysis, the data were presented as geometric mean, mean ± standard deviation, and assessed using analysis of variance (ANOVA), followed by the Bonferroni post hoc test, using GraphPad Prism 9.0. Significant differences were considered when *p* < 0.05.

## 5. Conclusions

Based on the execution of this study, it was observed that only the thiadiazine-derived compound, specifically IJ28, demonstrated antibacterial activity throughout the investigation. Molecular docking analysis reinforced these findings, revealing that thiadiazine derivatives, particularly IJ28, and CCCP share common interactions with residues, suggesting a significant affinity for the NorA protein. These results support the conclusion that the evaluated compounds modulate resistance to norfloxacin by inhibiting the NorA protein, highlighting their promising potential for application as antibacterial agents.

The results highlight the need for further research to understand the efflux mechanisms and the interaction of these compounds with bacterial resistance, aiming at the development of new therapeutic strategies against multidrug-resistant infections.

## Figures and Tables

**Table 1:** Molecular docking results with the NorA efflux pump.

Compounds	Binding Energy (Kcal/mol)	Inhibition Constant (µM)	Ligand Efficiency
**IJ26**	−5.30	129.52	−0.25
**IJ27**	−5.46	99.54	−0.27
**IJ28**	−5.55	84.96	−0.28
**IJ29**	−5.43	104.31	−0.27
**CCCP**	−4.63	403.17	−0.33

Ligand efficiency is obtained by dividing the binding energy by the number of non-hydrogen atoms.

**Table 2:** Interactions identified from IJ28 and CCCP and their respective distances.

Compounds	vdW	Hydrogen Bond	Hidrophobic	Electrostatic
**IJ28**	Arg310, Asn137, Asp307, Ile141, Phe341, Ser219, Thr223	Asn340 (1.77 Å) Glu222 (2.23 Å)	Phe140 (5.28 Å) Pro344 (5.29 Å)	-
**CCCP**	Ile136, Leu218, Pro344, Thr223, Thr336	Glu222 (1.74 Å) Ser219 (3.55 Å)	Phe140 (4.88 Å)	Arg310 (3.96 Å)

vdW: Van der Waals interactions (distance ≅ 5.0 Å).

## Data Availability

The data presented in this study are available on request from the corresponding author.
